# Cognitive and intellectual functioning in leukodystrophy patients: a systematic review

**DOI:** 10.1186/s13023-025-04083-7

**Published:** 2025-11-10

**Authors:** Wietske H. M. Grol, Marjo S. van der Knaap, Nicole I. Wolf, Gert J. Geurtsen

**Affiliations:** 1 Department of Child Neurology, Emma’s Children’s Hospital, Amsterdam UMC, Amsterdam, The Netherlands; 2 Amsterdam Leukodystrophy Center, Amsterdam Neuroscience, Cellular & Molecular Mechanisms, Amsterdam, The Netherlands; 3 Department of Integrative Neurophysiology, Center for Neurogenomics and Cognitive Research, Vrije Universiteit, Amsterdam, The Netherlands; 4 Department of Medical Psychology, Amsterdam Neuroscience, Amsterdam UMC, Amsterdam, The Netherlands; 5 Department of Medical Psychology, Amsterdam UMC – Location AMC, P.O. Box 22660, 1100 DD, Meibergdreef 9, Amsterdam, AZ 1105 The Netherlands

**Keywords:** Leukodystrophies, Cognitive functioning, Neuropsychological tests, Intelligence

## Abstract

**Background:**

‘Leukodystrophies’ encompass a group of genetic disorders affecting brain white matter, mostly characterized by neurological deterioration. Manifestations include mood changes, cognitive decline, motor dysfunction, and often early death. Underestimation of the cognitive decline may result in a lack of suitable interventions and adequate guidance. This systematic review aims to gain insight into available information on the cognitive and intellectual profile of leukodystrophy patients, providing a basis for further research.

**Methods:**

MEDLINE, EMBASE and PsychINFO were searched for studies examining processing speed, attention, memory, language, social cognition, visuospatial functioning and construction, and intelligence in patients with any type of confirmed leukodystrophy utilizing standardized neuropsychological tests. The included studies were analyzed to determine whether there are similarities among the various types of leukodystrophies and their associated cognitive and intellectual profiles. Due to significant variability in neuropsychological assessments, data synthesis was performed narratively rather than via meta-analysis.

**Results:**

Thirteen group studies and twenty case studies were included, with most group studies rated as moderate to high quality. On group level, cognitive and intellectual functioning varied widely between different leukodystrophy types. Intelligence and information processing speed were most frequently affected. A consistent finding was that the age of onset significantly influenced cognitive outcomes, with childhood-onset leukodystrophies generally associated with earlier and greater cognitive and intellectual impairments than adult-onset leukodystrophies.

**Conclusion:**

Although intelligence and cognition are obviously affected in the course of leukodystrophies, information processing speed being impaired relatively frequently, the specific profiles of cognitive and intellectual impairment are currently unclear, while such insight is crucial for proper guidance. Well-designed future studies are needed to establish a clearer cognitive and intellectual profile in leukodystrophies, their subtypes and during their disease course. Results will be the basis for suitable guidelines for testing and set the foundation for development of appropriate outcome measurements for therapy trials.

**Supplementary information:**

The online version contains supplementary material available at 10.1186/s13023-025-04083-7.

## Introduction

‘Leukodystrophies’ are genetic, mostly progressive brain disorders that specifically affect the white matter of the central nervous system (CNS) [1]. There are many different leukodystrophies with different underlying genetic defects [2]. Onset can be at any age, from prenatal life to senescence. The overall incidence of leukodystrophies is estimated at 1 in 4733 live births and differs for the different types of leukodystrophies [3]. Leukodystrophies are characterized by motor, cognitive and intellectual problems. A broad spectrum of motor signs may develop, such as spasticity, ataxia, movement disorders, and swallowing dysfunction [4]. Epilepsy and decreased vision due to optic atrophy may occur as well.

Currently, motor dysfunction receives most attention regarding characterization of patient problems and treatment, while the cognitive and/or intellectual deficits are neglected in our experience. As a result, little is known about these types of dysfunction, and little is done. The term “cognition” describes the mental processes, such as perception, memory, and problem-solving, that are involved in acquiring, storing and evaluating information. Intelligence is the ability to use these cognitive processes to reason, learn, and adjust to new situations. While cognition covers the more detailed mechanisms of thinking, intelligence measures the overarching capacity to use those mechanisms effectively [5]. Without a clear insight into the cognitive and intellectual profile of patients with a leukodystrophy, guidelines for managing the patient’s impairments remain lacking.

The aim of the current review is to systematically investigate the current knowledge of cognitive and intellectual functioning in patients with leukodystrophies in order to answer the following questions: (1) Is there a distinct profile of cognitive and intellectual dysfunction in leukodystrophy patients? and (2) Differ the profiles of cognitive and intellectual dysfunction between different leukodystrophies?

## Method

The current systematic review was conducted following the guidelines of the Preferred Reporting Items for Systematic Reviews and Meta-Analyses (PRISMA) method [6], see Additional file 1.

### Search strategy

**Fig. 1:**
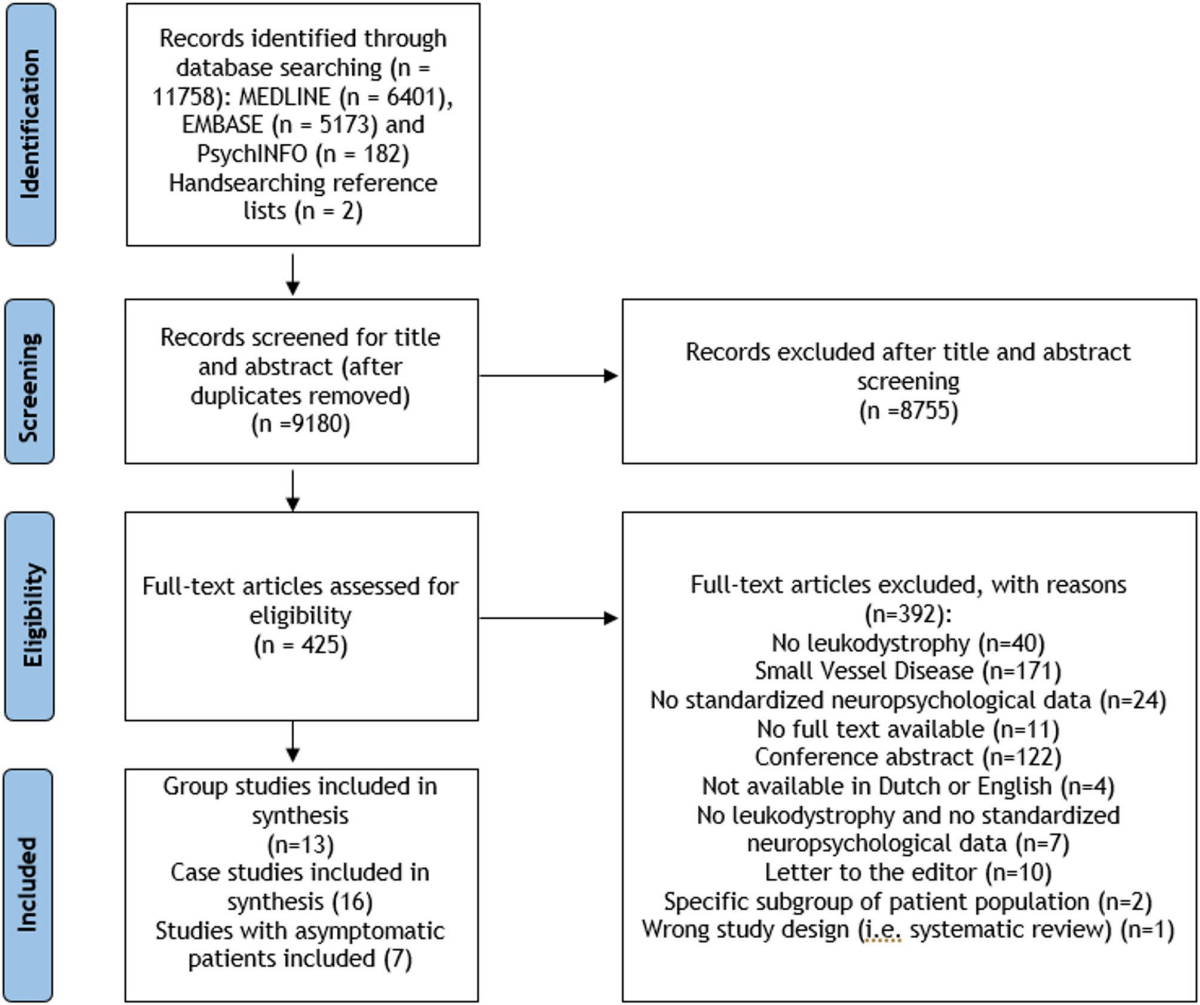
PRSIMA flow diagram. Flow diagram of the study selection process [57]. *Notes*. Three case studies consisted of both symptomatic patients and asymptomatic individuals with a leukodystrophy diagnosis; these studies are used in both the case and asymptomatic group

An independent literature search was conducted across the electronic databases MEDLINE, EMBASE and PsychINFO on 7 December 2023. The search strategy used PubMED MESH terms and was subsequently adapted for Embase and PsychINFO. The following search terms among others were used: ‘leukodystrophy’, ‘leukodystrophies’, ‘metachromatic leukodystrophy’, ‘adrenoleukodystrophy’, ‘vanishing white matter’, ‘Alexander disease’, ‘cognition’, ‘cognitive functioning’, ‘neuropsychological functioning’, ‘memory’, ‘intelligence’ and ‘processing speed’. For the detailed search strategy, see Additional file 2. Additionally, reference lists of included papers and (systematic) reviews regarding leukodystrophies and cognitive functioning were hand searched. All papers were uploaded in Rayyan and automatically deduplicated. An updated literature search, using the original search strategy, was conducted for the period from December 7, 2023, to June 6, 2025. This yielded approximately 1250 new records in MEDLINE, 844 in EMBASE and 16 in PsychINFO, which were screened for relevance. No additional studies meeting the inclusion criteria were identified. This information is integrated in Fig. 1.

### Eligibility criteria

Title and abstract screening was independently performed by WHMG and GJG. Subsequently, a full text analysis for eligibility was performed by WHMG. Discrepancies or difficulties were resolved by consensus and/or discussed with a third expert, if necessary (MSvdK).

Studies were included if they investigated patients with a leukodystrophy diagnosis, confirmed by metabolic testing, genetic analysis and/or MRI. Although genetically determined ‘Small Vessel Diseases’ (SVDs) cause a leukoencephalopathy and can be considered leukodystrophies, all SVD subtypes were excluded because of the frequent occurrence of infarcts and hemorrhages, which can also interfere with cognitive functioning. Regarding adrenoleukodystrophy (ALD), this review focused on cerebral ALD (CALD) only; the phenotypes ‘Addison Disease Only’ and ‘Adrenomyeloneuropathy’ were excluded due to their lack of brain white matter involvement [7–10]. Most leukodystrophies lead to diffuse cerebral white matter involvement, although in many leukodystrophies the frontal white matter degeneration is relatively severe. An exception is CALD, in which the two subtypes accounting for most patients have either parieto-occipital or frontal preferential involvement. Because of the potential differential impact on cognitive and intellectual function, we considered those subtypes separately.

For inclusion, studies were required to provide results from standardized neuropsychological tests with corresponding norms, examining a cognitive domain. Studies only evaluating overall cognitive status, using screeners like the Montreal Cognitive Assessment (MoCA) or Mini-Mental State Examination (MMSE), and studies only reporting aggregated scores of test batteries were excluded. Studies solely reporting cognitive functioning after allogenic Hematopoietic Stem Cell Transplantation (HSCT) were also excluded, as our focus was on investigating the cognitive profile without the influence of HSCT treatment. Regarding studies investigating the cognitive and/or intellectual profile before and after HSCT, only the baseline data were used. The majority of past studies investigating cognitive functioning in leukodystrophy patients primarily focused on intelligence, a prognostic parameter used before undergoing HSCT [11]. It was therefore expected to find multiple studies solely investigating intelligence, and those studies were included.

Full-text, original papers of any publication year written in English were included. Meta-analyses and systematic reviews were excluded. High quality group studies were preferred. Given the relatively low incidence of leukodystrophies, it was expected that more case studies than group studies had been conducted. Case studies meeting the inclusion criteria were to be summarized generally, and an overview of the study characteristics and cognitive outcomes were to be included in Additional file 3 and 4. Studies solely investigating clinically asymptomatic individuals with a leukodystrophy diagnosis were to be included as well, because differences in cognitive and intellectual functioning between asymptomatic individuals and symptomatic patients were expected [12].

### Data extraction

Data on study characteristics (i.e. sample size, study design), patient characteristics (i.e. age, sex, age at onset, criteria for diagnosis, including gene mutated) and cognitive/intellectual outcomes were extracted from the included studies by WHMG. The diagnosis was considered particularly important, as different leukodystrophies have a different clinical presentation and disease course. Age at presentation was included in the data extraction to account for differences in rate of disease progression for different ages of presentation: earlier presentation is typically associated with a more rapid disease course than later presentation. The following subtypes based on ages of presentation were distinguished in metachromatic leukodystrophy (MLD): late-infantile (onset before 2.5 years), juvenile (2.5 till 16 years) and adult (from 16 years on) [13]. In CALD these were: childhood (3 till 10 years), adolescent (11 till 20 years) and adult (from 21 years on) [14]. In Alexander disease (AxD) neonatal (onset in first month after birth), infantile (before 2 years), juvenile (2 till 12 years) and adult (from 12 years on) subtypes were distinguished [15].

Authors were contacted if standardized neuropsychological test results were mentioned but not described in detail in the paper. If they did not respond within 6 weeks and the described data were not sufficient for inclusion, the paper was excluded.

All neuropsychological (sub-)tests were categorized into different cognitive domains (i.e. memory, information processing speed, attention/executive functioning, language, visuoconstruction, visuospatial functioning and social cognition) [16, 17]. Test scores and/or a cognitive domain below the second percentile (i.e. z-scores ≤2.1) were described as impaired [16]. For longitudinal designs, only the baseline measurement was included to avoid the confounding effects of therapy.

### Risk of bias assessment

Risk of bias assessment was performed by WHMG and verified by GJG. The critical appraisal checklist of the Joanna Briggs Institute (JBI) was used for cross-sectional studies. As in the pre- and post-HSCT studies only the baseline data were used, we treated them as cross-sectional studies, and quality was assessed with the JBI.

The Scottish Intercollegiate Guidelines Network (SIGN) quality appraisal checklist was used for case-control studies. Although the JBI and SIGN tools were developed to assess study quality in qualitative aspects, previous systematic reviews introduced a quantitative approach. Each question of the JBI and SIGN checklist includes four options: yes, no, unclear/can’t say, and not applicable/does not apply. A score of one was given to the answer yes or not applicable/does not apply, and a score of zero was given to the other answers. Studies with a JBI score higher than 70% of the maximum score (8 points for JBI and 11 points for SIGN-checklist) were classified as having a high quality, those with a score between 50 and 70% as having a medium quality, and those with a score less than 50% as having a low quality [18, 19].

### Data synthesis

The neuropsychological and intelligence tests applied and the quality of the report of the results were evaluated for all included papers. If several papers investigated the same cognitive function using comparable tests and the study quality was adequate, a meta-analytic approach with random-effects model was planned. If a quantitative approach was not feasible due to the heterogeneity between studies, a narrative approach was to be performed.

All leukodystrophies included in this review are progressive. Assessments of cognitive and intellectual functions were done at a certain stage after the patients presented and were diagnosed. Results were therefore impacted to a high degree by the disease stage. Typically, patients are examined relatively early in the disease course, when decisions on HSCT or other therapeutic measures still have to be taken. Results in this systematic review were interpreted keeping this in mind.

## Results

### Study selection

The original database search identified 9646 records. After removal of duplicates, the remaining 7070 records were screened. Hand-searching reference lists resulted in two extra papers. Twelve authors were contacted to ask for standardized neuropsychological test results and/or additional information, not provided in their papers [20–31]. From three authors, no working e-mail address was found [27, 28, 31]. Four authors responded within the set period of six weeks and their papers were included [20, 21, 23, 25]. From two authors, one did not answer the e-mail [22] and one was not able to provide the questioned data [24], but their published data was still sufficient for inclusion in the review. Finally, 13 group studies were retained (Table 1), including 5 cross-sectional studies [12, 15, 20, 23, 25, 32], 7 longitudinal studies on HSCT [21, 22, 24, 33–36], and one case-control study [12]. Reasons for exclusion are reported in Fig. 1. The study characteristics and results of the 16 case studies [29, 37–51] and 7 studies on asymptomatic individuals [39, 49, 52–56] are presented in the Additional files 3, 4, 5 and 6.

### Study characteristics

The group studies sample consisted of 244 patients with a confirmed leukodystrophy (i.e. CALD (*N* = 170), MLD (*N* = 61), AxD (*N* = 8) and adult-onset leukoencephalopathy with axonal spheroids and pigmented glia (ALSP) (*N* = 5)) (Table 1). The sample size varied from four to 62. Four studies included only children [12, 20, 22, 36], one study included only adults [23], five studies included both children and adults [15, 32–35] and three studies did not report the mean age and/or age range [20, 24, 25]. Overall, the age range was between two and 62 years. Three studies used age- and sex-matched control subjects [12, 32, 33]. However, in two out of these three studies [32, 33] no cognitive and/or IQ data of the control subjects was available, so a comparison could not be made. Of 7 studies, baseline data before HSCT was used [21, 22, 24, 33–36].

The case studies sample consisted of 21 patients with a confirmed leukodystrophy (i.e. CALD (*N* = 4), MLD (*N* = 8), AxD (*N* = 4), ALSP (*N* = 1), Krabbe Disease (KD) (*N* = 3) and Vanishing White Matter (VWM) (*N* = 1)) (Additional file 3). The studies on asymptomatic individuals consisted of 103 individuals with a confirmed leukodystrophy but without clinical symptoms (i.e. CALD (*N* = 100) and MLD (*N* = 3)).

**Table 1:** Study characteristics of the included group studies

Author (Year)	Study Design	N [P]/[C]	Mean Age Y:M (Range)	Sex F:M	Diagnosis based on	Mean age of onset Y:M (range) Distribution subtypes
Beschle et al., (2020) [33]	Cross-sectional	47 [9]/[35]	11:6 (5–18:2)	8:4	Genetic analyses	8:0 (4:3–13:1) 3 EJ;6 LJ
Van Rappard et al., (2018) [34]	Cross-sectional	4 [4]/[0]	18:6 (12–27)	2:2	Enzyme/Genetic/MRI analyses	14:9 (8–26) 2 JUV.;2 AD
Strölin et al., (2017) [20]	Cross-sectional	25 [25]/[0]	10:5 (NR)	11:14	Enzyme/MRI analyses; Increased sulfatide excretion in urine	8:3 (NR) 25 JUV.
Tillema et al., (2015) [35]	Cross-sectional	40 [20]/[20]	13:7 (2.0–35.3)	15:5	NR	NR 3 LI;11 JUV.;6 AD
Bougnères et al., (2021) [23]	Cross-sectional	4 [4]/[0]	6:5 (4:4–7:5)	0:4	VLCFA assay/Genetic/MRI analyses	4:9 (3:5–6:3) 4 childhood
Peters et al., (2004) [36]	Cross-sectional	32 [32]/[0]	≤19 years	0:32	VLCFA assay/Genetic/MRI analyses	≤19 years, further details NR
Pierpont et al., (2017) [22]	Cross-sectional	62 [62]/[0]	8:4 (4:0–16:0)	0:62	NR	NR 62 Childhood/Adolesecent
Riva et al., (2000) [13]	Case-Control	15 [7]/[8]	8:4 (6:1–10:5)	0:7	VLCFA assay/MRI	NR 15 childhood
Schäfer et al., (2021) [26]	Cross-sectional	41 [41]/[0]	NR	0:41	NR	NR 41 Adult
Shapiro et al., (2000) [25]	Cross-sectional	12 [12]/[0]	NR	0:12	NR	7:0 (3.7–9.3) 12 Childhood
Suzuki et al., (2000) [37]	Cross-sectional	4 [4]/[0]	8:6 (6:6–10:2)	0:4	VLFCA assay/MRI analyses	8:3 (6:3–10:1) 4 Childhood CALD
Rush et al., (2023) [24]	Cross-sectional	5 [5]/[0]	42:2 (37–51)	3:2	Genetic analyses	NR 5 AD
Zampini et al., (2023) [16]	Cross-sectional	8 [8]/[0]	11:0 (5–23)	4:4	Genetic analyses	0:11 (0:5–0:18)

*Notes. N* Sample size*, P* Patients, *C* Control subjects, *Y* Years*, M* Months*, F* Female*, M* Male, *EJ* Early Juvenile, *LJ* Late Juvenile, *AS* Asymptomatic, *LI* Late Infantile, *JUV* Juvenile, *AD* Adult, *NR* Not reported, *VLCFA* Very long chain fatty cids

### Risk of bias assessment

Six group studies were of high quality [12, 21, 23–25, 32]. All other group studies were of moderate quality [15, 20, 22, 33–36]. The risk of bias assessments are provided in Additional file 7.

The most frequent quality issue was the lack of detailed description of the inclusion and exclusion criteria in eight out of thirteen studies [20, 22–24, 33–36]. Hence, a risk for selection bias cannot be excluded as details such as disease severity were unknown. Only three studies described confounding factors such as psychiatric disorders, age of onset and disease severity and controlled for these [12, 21, 25]. This lack of consideration for confounding factors in the majority of the studies might represent an issue in interpreting the results. There was considerable variability in utilized neuropsychological tests across studies, while intelligence tests were generally consistent. Due to this variability, the lack of considering and controlling for confounding factors, and the moderate quality of half of the included studies, a quantitative meta-analytic approach was not feasible. A systematic, narrative approach was utilized for the current review.

### Cognitive outcome

Detailed results of the neuropsychological tests are provided in Table 2.

**Table 2:** Results of neuropsychological and intellectual assessment reported in the included group studies

Author (Year)	Tests	Memory	Information Processing Speed	Attention/Executive functioning	Language	Visuospatial functioning & construction	Intelligence
**Metachromatic leukodystrophy (MLD)**							
Beschle et al., 2020 [33] *Juvenile MLD*	WISC-III/V WAIS-III/V K-ABC-I/II	NI	NI	NI	NI	NI	Not impaired (FSIQ = 84)
Van Rappard et al., (2018) [34] *Juvenile and Adult MLD*	WISC-III WAIS-III	NI	NI	NI	NI	NI	Patient 1 (*JUV*): Not impaired (PIQ = 81, VIQ = 111, TIQ = 92) Patient 2 (*JUV*): **Impaired (TIQ = 55)** Patient 3 (*JUV*): **Impaired (TIQ = 60)** Patient 4 (*AD*): Not impaired (TIQ = 72)
Strölin et al., 2017 [20] *Juvenile MLD*	WISC WAIS K-ABC	NI	NI	NI	NI	NI	Not impaired (Mean FSIQ = 79;(SD = 18))
Tillema et al., 2015 [35] Late infantile and Juvenile MLD	WISC WPPSI	NI	NI	NI	NI	NI	**Impaired** **(FSIQ = 67 (61–94**))
Tillema et al., 2015 [35] *Adult MLD*	WAIS-III	NI	NI	NI	NI	NI	Not impaired (FSIQ = 87 (53–92))
**Cerebral Adrenoleukodystrophy (CALD)**							
Bougnères et al., 2021 [23] *Childhood CALD*	WISC-III WPPSI-R	NI	NI	NI	NI	NI	Patient 1: Not impaired (VIQ = 103, PIQ = 111) Patient 2: Not impaired (VIQ = 108, PIQ = 99) Patient 3: Not impaired (VIQ = 98, PIQ = 103) Patient 4: Not impaired (VIQ = 114, PIQ = 104)
Peters et al., 2004 [36] *Childhood and/or Adolescent CALD* *Parieto-Occipital*	WISC-III WPPSI	NI	NI	NI	NI	NI	Not impaired (PIQ = 96 (45–122.5);VIQ = 99 (59–127))
Peters et al., 2004 [36] *Childhood and/or Adolescent CALD* *Frontal*	WISC-III WPPSI	NI	NI	NI	NI	NI	Not impaired (PIQ = 92 (61–100);VIQ = 89 (70–107))
Pierpont et al., (2017) [22] *Childhood and Adolescent CALD*	WISC III/IV WAIS III/IV	NI	Processing speed Not impaired (z = −0.7, SD = 18.63)	Working memory Not impaired (z = −0.7, SD = 16.04) Perceptual reasoning Not impaired (z = −0.3, SD = 19.26)	Verbal comprehension Not impaired (z = −0.4, SD = 17.84)	Perceptual reasoning Not impaired (z = −0.3, SD = 19.26)	NR
Riva et al., 2000 [13] *Childhood CALD*	WISC RPM WFT semantic BNT SCT BBT ITPA	ITTPA Auditory sequential memory: Not impaired (*z* = −0.89, *SD* = 0.63) Visual sequential memory: **Impaired (z = −3.17, SD = 1.45)**	Coding WISC **Impaired (z = −2.45, SD = 0.70)** Object assembly WISC **Impaired (z = −2.43, SD = 0.47)**	Mazes **Impaired (z = −2.1, SD = 0.86)**	WFT semantic **Impaired (z = −2.1, SD = 1.41)** BNT **Impaired (z = −18.98, SD = 15.06)**	SCT Not impaired (*z* = −1.75, *SD* = 1.85) BBT Not impaired (*z* = −1.54, *SD* = 0.94)	WISC FIQ: **Impaired (z = −2.28, SD = 0.76)** VIQ: Not impaired (*z* = −1.32, *SD* = 0.95) PIQ: **Impaired (z = −2.89, SD = 0.73)** RPM Not impaired (≥50^th^ percentile)
Schäfer et al., 2021 [26] *Adult CALD* *Parieto-Occipital*	MWT LPS-3 D2 WAFS AVLT VLT NVLT CORSI SCWT 5-Point Test SDMT RWT	AVLT Verbal encoding: Not impaired (*T* = 43.4, *SD* = 11.6) Verbal retrieval: Not impaired (*T* = 45.0, *SD* = 9.4) VLT Not impaired (*T* = 45.8, *SD* = 10.0) NVLT Not impaired (*T* = 45.1, *SD* = 10.1) CORSI Not impaired (*T* = 52.5, *SD* = 13.2)	SDMT Not impaired (*T* = 43.6, *SD* = 11.2)	D2 Not impaired (*T* = 36.5, *SD* = 9.7) WAFS Not impaired (*T* = 42.2, *SD* = 10.8) Stroop RI Not impaired (*T* = 49.7, *SD* = 8.4) Stroop Not impaired (*T* = 54.0, *SD* = 8.6) 5-POINT Not impaired (*T* = 52.1, *SD* = 11.2)	RWT Phonemic Not impaired (*T* = 40.6, *SD* = 11.4) RWT Semantic Not impaired (*T* = 40.2, *SD* = 11.8)	NI	MWT Verbal IQ: Not impaired (99.8, *SD* = 12.0) LPS-3 Non-verbal IQ: Not impaired (108.7, *SD* = 14.5)
Schäfer et al., 2021 [26] *Adult CALD* *Frontal*	MWT LPS-3 D2 WAFS AVLT VLT NVLT CORSI SCWT 5-Point Test SDMT RWT	AVLT Verbal encoding: Noy impaired (*T* = 39.3, *SD* = 2.1) Verbal retrieval: Not impaired (*T* = 46.0, *SD* = 10.5) VLT Not impaired: (*T* = 47.7, *SD* = 11.7) NVLT Not impaired: (*T* = 56.3, *SD* = 7.6) CORSI Not impaired: (*T* = 46.0, *SD* = 2.0)	SDMT Not impaired (*T* = 48.6, *SD* = 14.3)	D2 Not impaired (*T* = 51.0, *SD* = 15.6) WAFS Not impaired (*T* = 52.3, *SD* = 7.6) Stroop RI Not impaired (*T* = 46.3, *SD* = 4.0) Stroop NI Not impaired (*T* = 45.7, *SD* = 1.2) 5-POINT Not impaired (*T* = 51.8, *SD* = 10.9)	RWT Phonemic Not impaired (*T* = 38.2, *SD* = 11.7) RWT Semantic Not impaired (*T* = 39.7, *SD* = 8.5)	NI	MWT Verbal IQ: Not impaired (106.0, *SD* = 16.5) LPS-3 Non-verbal IQ: Not impaired (109.6, *SD* = 11.5)
Shapiro et al., 2000 [25] *Childhood CALD*	WISC WPPSI-R	NR	NR	NR	NR	NR	Not impaired: Mean Verbal IQ = 93 Mean Performance IQ = 95
Suzuki et al., 2000 [37] *Childhood CALD*	WISC	NI	NI	NI	NI	NI	Patient 1**: Impaired (FSIQ = 60)** Patient 2: **Impaired (VIQ = 74**; **PIQ = 61**) Patient 3: NI because severe visual, speech and mental disturbances Patient 4: NI because severe mental and gait disturbances
**Adult-onset Leukoencephalopathy with axonal Spheroids and Pigmented glia (ALSP)**							
Rush et al., 2023 [24]	DRS-2 - Attention WAIS-IV Digit Span TMT/DKEFS TMT SCWT/DKEFS CWT DRS-2 Conceptualization DRS-2 Initiation BNT COWAT/DKEFS letter/semantic fluency DRS-2 construction WAIS-V Block Design DRS-2 Memory CVLT WMS-IV - LM 1 WMS-IV - LM 2	z-scores consists of DRS memory; CVLT; LM 1 + 2 Patient 1: Not impaired (*z* = −1) Patient 2: Not impaired (*z* = −1.9) Patient 3: Not impaired (*z* = −0.26) Patient 4: Not impaired (*z* = −1.47) Patient 5: Not impaired (*z* = −0.74)	z-scores consists of TMT/DKEFS A; Stroop/DKEFS Word + Color Patient 1: **Impaired (z = −3)** Patient 2: **Impaired (z = −2.89)** Patient 3: Not impaired (*z* = −1.34) Patient 4: **Impaired (z = −3)** Patient 5: Not impaired (*z* = −0.34)	z-scores attention consists of DRS attention; digit span z-scores EF consists of TMT/DKEFS B/4; DRS initiation; DRS conceptualization; Stroop/DKEFS color-word Attention Patient 1: Not impaired (*z* = −1) Patient 2: Not impaired (*z* = −1) Patient 3: Not impaired (*z* = −1.17) Patient 4: Not impaired (*z* = −1.33) Patient 5: Not impaired (*z* = −0.67) EF Patient 1: Not impaired (*z* = −1.89) Patient 2: Not impaired (*z* = −2) Patient 3: Not impaired (*z* = −0.33) Patient 4: Not impaired (*z* = −1.89) Patient 5: Not impaired (*z* = 0)	z-scores consists of COWAT semantic/letter; BNT Patient 1: NR (missing BNT) Patient 2: NR (missing BNT) Patient 3: Not impaired (*z* = −0.22) Patient 4: **Impaired (z = −2.11)** Patient 5: Not impaired (*z* = 0.56)	z-scores consists of DRS construction; WAIS-IV Block design Patient 1: Not impaired (*z* = −0.84) Patient 2: Not impaired (*z* = −0.67) Patient 3: Not impaired (*z* = −0.67) Patient 4: Not impaired (*z* = −0.84) Patient 5: Not impaired (*z* = −0.17)	NI
**Alexander disease (AxD)**							
Zampini et al., 2023 [16] *Infantile AxD*	Subtests of BVN; STM forward STM backward Word recall Labeling Syntactic comprehension Phonemic fluency Categorical fluency	STM forward Patient 1: **Impaired (z = −3)** Patient 2: **Impaired (z = −2.5)** Patient 3: **Impaired (z = −2.8)** Patient 4: **Impaired (z = −3.9)** STM backward Patient 1: Not impaired (*z* = −1.2) Patient 2: Not impaired (*z* = −1.1) Patient 3: **Impaired (z = −4.5)** Patient 4: **Impaired (z = −4.6)** Word recall Patient 1: Not impaired (*z* = −1.8) Patient 2: **Impaired (z = −2.1)** Patient 3: **Impaired (z = −2.5)** Patient 4: **Impaired (z = −3)**	NI	NI	Labeling Patient 1: Not impaired (*z* = 1.1) Patient 2: Not impaired (*z* = −2) Patient 3: Not impaired (*z* = −0.8) Patient 4: Not impaired (*z* = 0.6) Syntactic Comprehension Patient 1: Not impaired (*z* = −1.9) Patient 2: **Impaired (z = −3.1)** Patient 3: Not impaired (*z* = −1.2) Patient 4: **Impaired (z = −3.7)** Phonemic fluency Patient 1: Not impaired (*z* = −1) Patient 2: **Impaired (z = −2.9)** Patient 3: **Impaired (z = −2.9)** Patient 4: Impaired (*z* = −2.7) Categorical fluency Patient 1: Not impaired (*z* = −1.4) Patient 2: Impaired (*z* = −3.1) Patient 3: Impaired (*z* = −2.2) Patient 4: Impaired (*z* = −3)	NI	NI

*WISC* Weschler Intelligence Scale for Children, *WAIS* Weschler Adult Intelligence Scale, *K-ABC* Kaufman Assessment Battery for Children, *NI* Not Investigated, *FSIQ* Full Scale Intelligence Quotient*, JUV* Juvenile, *PIQ* Performance Intelligent Quotient, *VIQ* Verbal Intelligence Quotient, *TIQ* Total Intelligence Quotient, *AD* Adult, *SD* Standard Deviation, *WPPSI* Weschler Preschool and Primary Scale of Intelligence, *NR* Not Reported, *RPM* Raven Progressive Matrices, *WFT* Word Fluency Test, *BNT* Boston Naming Test, *SCT* Street Completion Test, *BBT* Beery Buktenica Test, *ITPA* Illinois Test for Psycholinguistic Abilities, *MWT* Multiple Choice Vocabulary Test, *LPS-3* Performance Test System - Subtest 3, *WAFS* Attention Functions Battery, *AVLT* Auditory Verbal Learning Test, *VLT* Verbal Learning Test, *NVLT* Non-Verbal Learning Test, *CORSI* Corsi Block-Tapping Test, *SCWT* Stroop Color Word Test, *SDMT* Symbol Digits Modalities Test, *RWT* Regensburger Word Fluency Test, *Stroop RI* Stroop Reading Interference, *Stroop NI* Stroop Naming Interference, *DRS-2* Mattis Dementia Rating Scale, *TMT* Trail Making Test, *CVLT* California Verbal Learning Test, *WMS* Weschler Memory Scale, *LM* Logical Memory, *BVN* Neuropsychological Assessment Battery for Developmental Age, *STM* Short Term Memory

Z-scores below the second percentile (i.e. z-scores ≤2.1) were described as impaired

#### Information processing speed

***CALD***

Two high quality studies investigating information processing speed within childhood CALD patients presented different results [12, 21]. A small group study of high quality, including seven childhood CALD patients, found impairments on group level in information processing speed [12]. Symptomatic childhood CALD patients performed significantly worse (*p* ≤ 0.05) than asymptomatic individuals, who showed no deficits [12]. By contrast, no impairments in information processing speed were found on group level in 58 childhood and adolescent CALD patients [21], but the large standard deviation (SD = 18.63) suggests that some individuals had difficulties in this domain. In 41 adult CALD patients, no impairments in information processing speed were found [25], and no significant differences in performance between the frontal and parieto-occipital groups. All in all, the results suggest information processing speed may be a more commonly early affected domain in childhood onset CALD, than in adult onset CALD where this domain is less or later affected or not at all.

***ALSP***

One high quality study including five ALSP patients, who were neurologically symptomatic and had a manifest leukodystrophy on MRI, investigated information processing speed. Impairments in information processing speed were found in three out of four tested patients, suggesting that information processing speed may be affected in ALSP [23].

#### Memory

***CALD***

Verbal memory was assessed in two high quality studies [12, 25] and no impairments were found on group level in 41 adult onset CALD [25] and in seven childhood onset CALD patients [12]. Impairments in non-verbal memory were found in seven childhood CALD patients [12], whereas 41 adult CALD patients (both parieto-occipital and frontal) did not show non-verbal memory impairments [25]. Regarding non-verbal memory, the seven symptomatic childhood CALD patients performed significantly worse (*p* ≤ 0.05) than eight asymptomatic individuals, who showed no deficits [12]. Considering the fact that all patients were neurologically symptomatic and had a manifest leukodystrophy on MRI, these mixed results suggest memory impairments are not an early or major issue in CALD, and rather an issue in childhood onset CALD than adult onset CALD.

***ALSP***

Verbal memory was investigated in one high quality study including five ALSP patients, who were neurologically symptomatic and had a manifest leukodystrophy on MRI, and no deficits were found [23], suggesting that memory is not prevalently affected in early stages of ALSP patients.

***AxD***

Verbal and non-verbal memory deficits were found in a small cross-sectional study of moderate quality, including eight infantile onset AxD patients with current ages of 5–23 years [15]. Considering the disease duration of these patients, they likely had advanced disease.

#### Attention/Executive functioning

***CALD***

Attention and executive functioning were assessed in three high quality studies [12, 21, 25]. Mixed results were found in childhood CALD. A small group study comprising seven childhood CALD patients indicated impairment on group level in this domain [12]. Symptomatic childhood CALD patients performed significantly worse (*p* ≤ 0.05) than asymptomatic children, who showed no deficits in this domain [12]. By contrast, a study of 51 childhood and adolescent CALD patients showed no impairments on group level, although the large standard deviation (SD = 16.04) suggests that some individuals did have impairments in this domain [21]. In 41 adult CALD patients, no impairments in attention and executive functioning were found [25]. These mixed results suggest that attention and executive functioning may be more commonly affected in early stages of childhood onset CALD, but less or later or not at all in adult onset AL.D.

*ALSP*

No impairment in attention and executive functioning was found in five adult ALSP patients [23], suggesting that attention and executive functioning impairments are not a severe issue in early stages of ALSP.

#### Language

***CALD***

Two high quality studies investigating receptive language in childhood and adolescent CALD patients [12, 21] reported different results. A small cross-sectional study consisting of seven childhood CALD patients [12] reported impairments in receptive language, whereas no impairments on group level were found in a larger sample of 59 childhood and adolescent CALD patients [21]. Expressive language was examined in childhood and adult CALD patients in two high quality studies [12, 25]. Impairments in expressive language were reported in seven childhood CALD patients [12], whereas no impairments were found in 41 adult CALD patients [25]. Symptomatic childhood CALD patients performed significantly worse (*p* ≤ 0.05) in both receptive as expressive language than asymptomatic children, who showed no deficits in this domain [12]. These mixed results suggest that language impairments may occur in childhood CALD patients, but not in adult onset CALD.

***ALSP***

In a small cross-sectional study [23], two out of three ALSP patients showed expressive language impairments. Impairments were only found on the speeded measures of language; without time pressure there were no impairments. The authors therefore deemed it likely that the found impairments were caused by slowed information processing speed rather than a language impairment per se. These results provide additional evidence, as described above, for information processing speed impairments as an early and major issue in ALSP patients.

#### Visuospatial functioning and construction

***CALD***

No impairments in two tests of visuospatial functioning and construction were found in seven childhood CALD patients [12]; the symptomatic patients performed significantly worse than the asymptomatic children on only one test (*p* ≤ 0.05). These limited results suggest that visuospatial functioning and construction impairments are not an early or major issue in childhood CALD patients.

***ALSP***

No impairments in visuospatial functioning and construction were found in five adult ALSP patients, measured with two tests, leading to the same conclusion as for CALD [23].

#### Intelligence

***MLD***

Impaired intelligence levels were found in two out of four studies [32, 34]. In a moderate quality study, IQ was impaired in two juvenile MLD patients (mean full scale intelligence quotient (FSIQ) = 58), while lower than average, but not impaired in one juvenile and one adult MLD patient (mean FSIQ = 82) [34]. A high quality study investigating eight late infantile and juvenile and five adult MLD patients showed impairments in late infantile and juvenile (mean FSIQ = 67; range 61–94), while lower than average but not impaired in the adult MLD patients (FSIQ = 87; range 53–92) [32]. The range in IQ scores (53–92) in the adult MLD patients indicates that there were impairments on individual level. By contrast, two moderate quality studies did find lower than average but not impaired intelligence levels in 34 juvenile MLD patients (FSIQ = 79, SD = 18 and FSIQ = 84) [20, 33]. The large standard deviation in the group study on 25 juvenile MLD patients indicates that there were impairments on individual level [20].

The results suggest that diminished (impaired or lower than average) intelligence levels are a common and early issue in MLD patients.

***CALD***

Impaired intelligence was detected in two out of six studies [12, 36]. A high-quality study with seven childhood CALD patients found impairments in FSIQ, revealing a discrepancy between a normal verbal IQ and an impaired perceptual IQ [12]. Additionally, symptomatic childhood CALD patients performed significantly worse in both verbal and perceptual intelligence (*p* ≤ 0.05) than asymptomatic children, who showed no deficits in intelligence [12]. A moderate quality study including four childhood CALD patients showed impaired intelligence levels in two out of four patients; two patients were not formally tested due to already severe visual, gait and cognitive impairment. On the other hand, four studies investigating intelligence in 16 childhood, 32 childhood and adolescent (details about age of onset were not reported, all patients were ≤19 years) and 41 adult CALD patients did not detect IQ impairments [22, 24, 25, 35]. However, the large range in IQ scores in the study investigating 32 childhood/adolescent CALD patients indicates that there were impairments on individual level (Table 2 for detailed results). These results suggest that diminished intelligence may be an early or major issue in CALD patients.

#### Social cognition

None of the included studies examined social cognition with standardized neuropsychological tests. Therefore, it remains unclear whether social cognition is impaired in leukodystrophy patients.

#### Case studies

In total 16 case studies investigating cognitive functioning in MLD, CALD, AxD, ALSP, VWM, and KD patients were included in this systematic review [29, 37–51]. Results were heterogeneous within and between the different types of leukodystrophies; detailed results can be found in Additional files 3 and 4.

#### Case and group studies

Across both group and case studies, variability in cognitive and intellectual functioning was observed between and within leukodystrophy subtypes, with diffuse forms such as MLD more commonly associated with impaired intelligence and broader cognitive deficits than more focal forms like early-stage CALD. Impairments in information processing speed emerged as a consistent and often early finding across multiple leukodystrophies, while other cognitive domains showed more heterogeneous patterns. Case studies largely supported the group-level findings and provided additional insight into underreported subtypes such as AxD and ALSP, reinforcing the association between disease type, extent of white matter involvement, and the severity and pattern of cognitive impairment (see Additional files 3 and 4).

#### Studies of asymptomatic individuals

In total nine studies investigating neuropsychological functioning in asymptomatic individuals with a leukodystrophy diagnosis were included in this systematic review [33, 37, 39, 44, 49, 53–56]. No cognitive impairment was detected in any of the measured domains within asymptomatic CALD, MLD and KD individuals. Detailed results can be found in Additional files 5 and 6.

## Discussion

### Main findings

This systematic review investigated cognitive and intellectual impairments in leukodystrophy patients, analyzed the specific cognitive domains affected, and aimed at gaining insight into the intellectual profile in these patients. Thirteen group studies were included and reviewed in detail. The quality of the included group studies was moderate to high. Sixteen case studies and seven studies investigating asymptomatic individuals with a leukodystrophy diagnosis were included in this review and described more superficially. This review revealed large differences in cognitive and intellectual functioning between and within various types of leukodystrophies (CALD, MLD, ALSP and AxD). In the leukodystrophy literature, the terms “cognitive” and “intellectual” functioning are often used interchangeably, reflecting the limited availability of detailed neuropsychological data. Intelligence quotient (IQ) scores, such as verbal IQ (VIQ), performance IQ (PIQ), and full-scale IQ (FSIQ), are frequently employed as broad indicators of cognitive function. While IQ scores provide global indices of intellectual ability, they inherently encompass components that overlap with domain-specific cognitive skills typically assessed through detailed neuropsychological testing. We acknowledge that conceptually, cognitive and intellectual functions are not entirely distinct constructs, and distinctions can be nuanced. Nonetheless, given the current state of the literature, using these terms in a complementary manner allows for a practical synthesis of the available data. Recognizing this nuance is important for accurately interpreting findings and understanding the cognitive profiles described in this patient population.

A major problem for this systematic review is the fact that the diseases investigated lead to progressive neurological impairment and that the studies contained patients in different stages of the disease, hampering comparison of the results of the different studies and answering our research questions. So, conclusions made need to be regarded with caution. It is likely that the assessments were performed relatively early in the disease and not in end-stage disease. Overall, it is clear from all studies that the age of onset has an important impact on the level of functioning and that childhood onset disease, being more rapidly progressive, is associated with more intellectual and cognitive deficits than adult onset disease.

Intelligence was investigated in both MLD and CALD patients. In the MLD patients, the average intellectual level was below average to low, while a proportion of the patients performed on an impaired intelligence level. By contrast, in the CALD patients the majority performed on an average intelligence level, with the minority of patients showing intellectual impairments. Case studies in juvenile and adult MLD and childhood CALD revealed intelligence impairments as well [36, 40, 49]. The differences between MLD and CALD could be explained by the fact that MLD is a diffuse white matter disease from onset, whereas CALD in the early stages most typically starts with involvement of either the parieto-occipital or frontal white matter only. In the group studies included in this review, intelligence was not tested in ALSP and AxD. However, two case studies on AxD investigated intelligence and showed impaired intellectual functioning [50, 51]. These results suggest intelligence levels are commonly diminished (impaired or lower than average) in patients with a diffuse leukodystrophy from a rather early stage.

Neuropsychological functioning was reviewed per cognitive domain and per leukodystrophy type (MLD, ALD, AxD and ALSP) yielding mixed results. As with intelligence, differences in cognitive functioning were found between and within the different leukodystrophy types. Overall, impairments in information processing speed were found in both childhood CALD patients and ALSP patients and appear to be relatively prevalent and early [12, 21, 23]. The case studies revealed information processing speed impairments in adult MLD, ALSP and juvenile KD as well [41, 43, 44]. Deficits in attention/executive functioning and language may also be affected early, especially in childhood onset disease, while deficits in visuospatial functioning and construction appeared less common. Non-verbal memory deficits were found in childhood CALD and AxD [12, 15]. In addition, case studies revealed both verbal and non-verbal memory deficits in ALSP and juvenile AxD [43, 51]. The other group studies did not find memory impairments. Overall, memory is possibly a less prevalent affected domain in early stages, but the results remain heterogeneous. Social cognition was not investigated at all. It therefore remains unclear whether leukodystrophy patients have impairments in their social competences.

Two high quality studies examined both intelligence and cognition in detail, demonstrating a relationship between the two. The findings indicate that impaired intelligence is associated with deficits across multiple cognitive domains [12]. Conversely, the results show that patients with normal intelligence levels do not exhibit cognitive impairments [25].

Regarding asymptomatic individuals with a leukodystrophy diagnosis, no cognitive or intellectual impairment was observed in the seven included studies (ALD, MLD and KD).

### Limitations

Drawing conclusions from these studies and comparison between them were complicated by several methodological limitations.

At the start of the study, we were aware of the fact that little information was available about cognitive function and intelligence in leukodystrophies, which our review confirmed. The number of studies per cognitive domain and per leukodystrophy type was low, with small sample sizes in the majority of studies. In addition, the number and types of neuropsychological tests used varied across studies and complicated comparison of results. The majority of studies used different paper-and-pencil tests per cognitive domain, however the Stroop Color Word test, the Boston Naming Test and the Phonemic and Categorical Fluencies were administered in multiple studies [12, 15, 23, 25]. One study used a computerized neuropsychological test battery for memory and language [15]. It is unclear whether in this study [15] all the requirements for reliable online testing were met [58]. Only the administered intelligence tests were the same across studies and could be compared. Some studies may not have used a sufficiently broad battery of tests to detect cognitive deficits, increasing the risk of underestimation. Unfortunately, multiple studies describing cognitive impairments in leukodystrophy patients could not be included in this review because standardized neuropsychological scores or used tests were not reported or not provided despite repeated requests [11, 59–61].

Due to the limited number of studies and the small sample sizes in most of these studies, there is a risk of bias. Additionally, the majority of the included group studies (eight out of thirteen) did not provide sufficient detail on the recruitment process, increasing the risk of selection bias [20, 22–24, 33–36]. Given the progressive nature of leukodystrophies, the disease stage very likely has important impact on cognitive and intellectual functioning, but in the majority of the included studies disease stage was not described, complicating the interpretation and hampering comparison of results. Furthermore, severely affected patients are typically not included for the neuropsychological and intelligence assessment, causing an underestimation of effects. Seven of the included studies are longitudinal HSCT studies, of which baseline data were used [21, 22, 24, 33–36]. Preserved cognitive and intellectual functioning is a predictor of successful HSCT outcomes [11]; therefore, these patients were mostly early in disease stage, and inclusion was probably selective and not representative of leukodystrophy patients at all stages. Additionally, confounding factors such as neurological or psychiatric disorders were not adequately addressed and/or accounted for in most of the studies.

### Implications for future research

To overcome some of the methodological issues described and to improve the knowledge of cognitive and intellectual functioning in leukodystrophies, well-designed and well-reported studies are needed, covering multiple cognitive domains and using a test battery with a wide range of well normed neuropsychological tests with multiple tests per domain, sensitive enough to identify subtle cognitive impairments. Preferably, tests should be suitable for serial repetition to determine the effects of disease progression. Important cognitive domains to investigate include information processing speed, attention, memory, language, visuospatial perception, executive functioning and social cognition. Information processing speed is especially important in patients with leukodystrophies, as white matter abnormalities have been associated with lower information processing speed [62–64]. Social cognition has not yet been investigated using neuropsychological tests, which is remarkable as earlier research with the VINELAND-3 battery, a subjective interviewing technique of caregivers, suggests early and pronounced impairment in this domain. Neuropsychological tests could help to see if there is a cognitive underpinning of these findings.

Future studies should describe the inclusion and exclusion criteria in more detail, including the age of onset and disease stage, reported clinical signs and symptoms, and severity of the abnormalities on brain MRI. Including patients with different disease stages will lower the risk of underestimation of cognitive and intellectual impairments that individuals with leukodystrophies already have and may develop. By controlling for age of onset and disease stage, it will be possible to draw conclusions regarding the cognitive and intellectual profile of leukodystrophy patients at different stages of these progressive diseases. Longitudinal studies will further enhance the knowledge of the course of the cognitive and intellectual functioning. Furthermore, cognitive outcomes should be reported in detail, including quantitative data from tests and their subtests (i.e. mean raw score, standard deviation, interquartile range, normative score and the percentage of patients performing on impaired level). This level of detail will allow for a more precise understanding of specific cognitive strengths and weaknesses, facilitate comparisons across studies, and support a more nuanced analysis of how different leukodystrophies impact various cognitive domains and their individual differences. Investigating intelligence alone is insufficient in this patient population, because intelligence tests are broad assessment tools that may not detect subtle changes over time or more specific cognitive impairments. Neuropsychological assessment using a comprehensive test battery may serve as a more reliable predictor of successful HSCT.

Another important limitation of the current literature is the lack of data on the impact of cognitive and intellectual functioning on quality of life, levels of independence in activities of daily living, and educational attainment. These factors are highly relevant for understanding the full burden of disease and for supporting clinicians in counseling families. As such information was not available in the studies included in this review, it represents a critical area for future research.

Accounting for the limitations in future research can be the basis for the development of outcome measurements for interventions such as HSCT and gene therapy. Insight in the neuropsychological and intellectual profile per leukodystrophy subtype will also be the basis for better understanding by the patient, caregivers, teachers and coworkers and consequently help creating a more suitable guidance plan. Eventually, these insights may help enhance the health-related quality of life of leukodystrophy patients in both early and late stages of the disease [65].

## Conclusions

This systematic review revealed that age of onset of leukodystrophies has an important impact on the level of functioning, and that childhood onset disease is associated with more intellectual and cognitive deficits than adult onset disease. These findings of the review also underscore the importance of considering the disease stage in future research and highlights the need for longitudinal studies that document disease progression in the same patients, allowing for a clearer understanding of the relationship between disease stage and outcome of installed therapies. Additionally, this review revealed that certain domains, particularly information processing speed, is affected early across multiple leukodystrophies, suggesting that processing speed may be an important sensitive outcome measure in future research. The social domain, which has been largely overlooked, is important to prioritize, especially given the findings in VWM that suggest early and severe impairment in social cognition. Furthermore, intelligence appears to be more affected in diffuse leukodystrophies than in more localized ones, suggesting that the relevance of intelligence as an outcome measure varies depending on the type of leukodystrophy. Well-designed future studies are needed to address these knowledge gaps and establish a clearer cognitive and intellectual profile in leukodystrophy patients. Results will be the basis for creating suitable guidelines and set the foundation for development of appropriate outcome measurements for available and emerging therapy trials and therapies.

## Electronic supplementary material

Below is the link to the electronic supplementary material.

Supplementary Material 1

Supplementary Material 2

Supplementary Material 3

Supplementary Material 4

Supplementary Material 5

Supplementary Material 6

Supplementary Material 7

## Data Availability

This is a systematic review. We have provided the search parameters and mesh terms (Table S1) whereby others can replicate the search and reproduce the findings. Actual tabulation of the reviewed papers and summaries of the papers are provided in this systematic review in Tables 2 and 3 and the supplementary information.

## Abbreviations

CALD: Cerebral Adrenoleukodystrophy
ALSP: Adult-onset Leukoencephalopathy with axonal Spheroids and Pigmented glia
AxD: Alexander Disease
CNS: Central Nervous System
HSCT: Hematopoietic Stem Cell Transplantation
IQ: Intelligence Quotient
JBI: Joanna Briggs Institute
KD: Krabbe Disease
MLD: Metachromatic Leukodystrophy
MMSE: Mini-Mental State Examination
MoCA: Montreal Cognitive Assessment
MRI: Magnetic Resonance Imaging
PRISMA: Preferred Reporting Items for Systematic Reviews and Meta-Analyses
SIGN: Scottish Intercollegiate Guidelines Network
SVD: Small Vessel Disease
VWM: Vanishing White Matter
