# Supplementary material for: Cognitive and intellectual functioning in leukodystrophy patients: a systematic review
Source: Orphanet J Rare Dis. 2025 Nov 10;20:570. doi: 10.1186/s13023-025-04083-7 (PMC12604172; doi:10.1186/s13023-025-04083-7)
Supplement: Supplementary file 2 — Supplementary Material 2 [file 13023_2025_4083_MOESM2_ESM.docx]

**Additional file 2.** Search strategies. Search strategies for MEDLINE, EMBASE and PsychINFO used in the current systematic review.

**Table 1. Search Strategy MEDLINE (7-12-2023)**

| **#** | **Search** | **Number of results** |
| --- | --- | --- |
| 1 | ((((((((((((((((((((((("metachromatic leukodystrophy") OR ("MLD")) OR ("Adrenoleukodystrophy")) OR ("ALD")) OR ("X-ALD")) OR ("XALD")) OR ("Vanishing White Matter")) OR ("VWM")) OR ("4H syndrome")) OR ("Alexander disease")) OR ("Adult onset dominant leukodystrophy")) OR ("ADLD")) OR ("Adult leukoencephalopathy with spheriods and pigmented glia")) OR ("ALSP")) OR ("Hereditary dominant leukoencephalopathy with axonal spheriods")) OR ("HDLS")) OR ("Pigmented orthochromatic leukodystrophy")) OR ("POLD")) OR ("CADASIL")) OR ("Cerebral Autosomal Dominant Arteriopathy with Subcortical Infarcts and Leukoencefalopathy")) OR ("Small vessel disease")) OR ("SVD")) OR ("Leukodystrophy")) OR ("Leukodystrophies")  **AND**  (((((((((((((((((((((((((((("Cognition") OR ("Cognitive functioning")) OR ("Neuropsychology")) OR ("Neuropsychological functioning")) OR ("Memory")) OR ("learning")) OR ("Forgetting")) OR ("Working memory")) OR ("Visual perception")) OR ("Visuospatial")) OR ("Perception")) OR ("Constructive functioning")) OR ("Attention")) OR ("Executive")) OR ("Executive functioning")) OR ("Problem solving")) OR ("Reasoning")) OR ("Planning")) OR ("Information Processing")) OR ("Processing speed")) OR ("Social cognition")) OR ("Cognitive control")) OR ("Decision making")) OR ("Shifting")) OR ("Concentration")) OR ("Language")) OR ("IQ")) OR ("Intelligence")) OR ("Intellect") | 5903 |
| 2 | (((((((((((((((((("metachromatic leukodystrophy") OR ("MLD")) OR ("Adrenoleukodystrophy")) OR ("ALD")) OR ("X-ALD")) OR ("XALD")) OR ("Vanishing White Matter")) OR ("4H syndrome")) OR ("Alexander disease")) OR ("ADLD")) OR ("ALSP")) OR ("HDLS")) OR ("Pigmented orthochromatic leukodystrophy")) OR ("POLD")) OR ("CADASIL")) OR ("Small vessel disease")) OR ("SVD")) OR ("Leukodystrophy")) OR ("Leukodystrophies")  **AND**  (((((((((((((((((((((((((((("Cognition") OR ("Cognitive functioning")) OR ("Neuropsychology")) OR ("Neuropsychological functioning")) OR ("Memory")) OR ("learning")) OR ("Forgetting")) OR ("Working memory")) OR ("Visual perception")) OR ("Visuospatial")) OR ("Perception")) OR ("Constructive functioning")) OR ("Attention")) OR ("Executive")) OR ("Executive functioning")) OR ("Problem solving")) OR ("Reasoning")) OR ("Planning")) OR ("Information Processing")) OR ("Processing speed")) OR ("Social cognition")) OR ("Cognitive control")) OR ("Decision making")) OR ("Shifting")) OR ("Concentration")) OR ("Language")) OR ("IQ")) OR ("Intelligence")) OR ("Intellect") | 5306 |

**Table 2. Search strategy EMBASE (7-12-2023)**

| **#** | **Search** | **Number of results** |
| --- | --- | --- |
| 1 | (((((((((((((((((("metachromatic leukodystrophy") OR ("MLD")) OR ("Adrenoleukodystrophy")) OR ("ALD")) OR ("X-ALD")) OR ("XALD")) OR ("Vanishing White Matter")) OR ("4H syndrome")) OR ("Alexander disease")) OR ("ADLD")) OR ("ALSP")) OR ("HDLS")) OR ("Pigmented orthochromatic leukodystrophy")) OR ("POLD")) OR ("CADASIL")) OR ("Small vessel disease")) OR ("SVD")) OR ("Leukodystrophy")) OR ("Leukodystrophies")  **AND**  (((((((((((((((((((((((((((("Cognition") OR ("Cognitive functioning")) OR ("Neuropsychology")) OR ("Neuropsychological functioning")) OR ("Memory")) OR ("learning")) OR ("Forgetting")) OR ("Working memory")) OR ("Visual perception")) OR ("Visuospatial")) OR ("Perception")) OR ("Constructive functioning")) OR ("Attention")) OR ("Executive")) OR ("Executive functioning")) OR ("Problem solving")) OR ("Reasoning")) OR ("Planning")) OR ("Information Processing")) OR ("Processing speed")) OR ("Social cognition")) OR ("Cognitive control")) OR ("Decision making")) OR ("Shifting")) OR ("Concentration")) OR ("Language")) OR ("IQ")) OR ("Intelligence")) OR ("Intellect") | 3593 |

**Table 3. Search strategy PsychINFO (7-12-2023)**

| **#** | **Search** | **Number of results** |
| --- | --- | --- |
| 1 | (((((((((((((((((("metachromatic leukodystrophy") OR ("MLD")) OR ("Adrenoleukodystrophy")) OR ("ALD")) OR ("X-ALD")) OR ("XALD")) OR ("Vanishing White Matter")) OR ("4H syndrome")) OR ("Alexander disease")) OR ("ADLD")) OR ("ALSP")) OR ("HDLS")) OR ("Pigmented orthochromatic leukodystrophy")) OR ("POLD")) OR ("CADASIL")) OR ("Small vessel disease")) OR ("SVD")) OR ("Leukodystrophy")) OR ("Leukodystrophies")  **AND**  (((((((((((((((((((((((((((("Cognition") OR ("Cognitive functioning")) OR ("Neuropsychology")) OR ("Neuropsychological functioning")) OR ("Memory")) OR ("learning")) OR ("Forgetting")) OR ("Working memory")) OR ("Visual perception")) OR ("Visuospatial")) OR ("Perception")) OR ("Constructive functioning")) OR ("Attention")) OR ("Executive")) OR ("Executive functioning")) OR ("Problem solving")) OR ("Reasoning")) OR ("Planning")) OR ("Information Processing")) OR ("Processing speed")) OR ("Social cognition")) OR ("Cognitive control")) OR ("Decision making")) OR ("Shifting")) OR ("Concentration")) OR ("Language")) OR ("IQ")) OR ("Intelligence")) OR ("Intellect") | 747 |
