# Supplementary material for: Cognitive and intellectual functioning in leukodystrophy patients: a systematic review
Source: Orphanet J Rare Dis. 2025 Nov 10;20:570. doi: 10.1186/s13023-025-04083-7 (PMC12604172; doi:10.1186/s13023-025-04083-7)
Supplement: Supplementary file 3 — Supplementary Material 3 [file 13023_2025_4083_MOESM3_ESM.docx]

**Additional file 3.** Study characteristics of the included case studies.

**Table 1. Study characteristics of the included case studies**

| **Author (Year)** | **N** | **Age Y:M** | **Gender** | **Criteria for diagnosis** | **Age of onset**  **(Y:M)**  **Subtype** |
| --- | --- | --- | --- | --- | --- |
| Cable et al., 2011; Pierson et al., 2008 | 1 | 8:NR | Male | MRI and urine sulfatide analysis | 8:NR  Juvenile |
| Johannsen et al., 2001 | 1 | 41:NR | Male | Neuropsychological assessment; MRI analysis; ARSA dein tficiency | NR  Adult |
| Shapiro et al., 1992 | 1 | 4:NR | Female | ARSA deficiency; MRI analysis | 0:8  Late infantile |
| Smith et al., 2010 | 1 | 23:NR |  | MRI analysis; ARSA deficiency; Sural nerve biopsy | 23:NR  Adult |
| Solders et al., 1998; Solders et al., 2014; | 3 | Patient 1: 28:NR  Patient 2: 17:6  Patient 2: 19:6 | Patient 1: Female  Patient 2: Female  Patient 2: Female | MRI analysis; ARSA deficiency; Neuropsychological assessment; Urine sulfatide analysis | Patient 1: 24:NR  Patient 2: 16:NR  Patient 3: 18:NR  Adult |
| Videbaek et al., 2021 | 1 | 23:NR | Female | MRI analysis; Neuropsychological assessment; ARSA deficiency; Genetic analysis | 18:NR  Adult |
| Malm et al., 1997 | 3 | Patient 1: 9:6  Patient 2: 6:0  Patient 3: 4:6 | Patient 1: Male  Patient 2: Male  Patient 3: Male | VLFCA evaluation; MRI analysis; Neuropsychological assessment | NR  Childhood Cerebral |
| Turco et al., 2018 | 1 | 9:NR | Male | MRI analysis; EEG; VLFCA, PCR and MLPA analysis | NR  Childhood Cerebral |
| Kirsch et al., 2021 | 2 | Patient 1: Tested from 6 to 12 years of age  Patient 2: Tested from 12 to 16 years of age | Patient 1: Male  Patient 2: Female | MRI and genetic analysis | Patient 1: 2:NR  Juvenile  Patient 2: 10:NR Juvenile |
| Restrepo et al., 2011 | 1 | Tested from 15 to 21 years of age | Male | MRI and genetic analysis; EEG; Neuropsychological assessment | 9:NR  Juvenile |
| Wilson et al., 2018 | 1 | 14:NR | Female | NR (‘Many diagnostics examinations’) | 5:NR  Juvenile |
| Trevisan et al., 2021 | 1 | 52:NR | Male | MRI and genetic analysis; EEG; | NR  Adult |
| Mateen et al., 2010 | 1 | 24:NR | Female | MRI and genetic analysis; Neuropsychological assessment | 23:NR  Adult |
| Krivit et al., 1998 | 3 | Patient 1: 11:NR  Patient 2: 8:NR  Patient 3: 2:NR | Patient 1: Female  Patient 2: Male  Patient 3: Male | Enzymatic assay; MRI analysis; Neuropsychological assessment | Patient 1: 5:NR  Juvenile  Patient 2: 7:NR (asymptomatic)  Patient 3: 2:NR (asymptomatic) |

*Notes. N* Sample size*, P* Patients, *Y* Years*, M* Months*, VLCFA* Very Long Chain Fatty Acids.
