# Supplementary material for: Cognitive and intellectual functioning in leukodystrophy patients: a systematic review
Source: Orphanet J Rare Dis. 2025 Nov 10;20:570. doi: 10.1186/s13023-025-04083-7 (PMC12604172; doi:10.1186/s13023-025-04083-7)
Supplement: Supplementary file 4 — Supplementary Material 4 [file 13023_2025_4083_MOESM4_ESM.docx]

**Additional file 4.** Results of neuropsychological and intellectual assessment reported in the included case studies.

**Table 2.** Results of neuropsychological and intellectual assessment reported in the included case studies

| **Author (Year)** | **Tests** | **Memory** | **Information Processing Speed** | **Attention/ Executive functioning** | **Language** | **Visuospatial functioning & construction** | **Intelligence** |
| --- | --- | --- | --- | --- | --- | --- | --- |
| **Metachromatic leukodystrophy (MLD)** | | | | | | | |
| Cable et al., 2011; Pierson et al., 2008  *Juvenile MLD* | NR which intelligence test was used | NI | NI | NI | NI | NI | **Impaired (FSIQ = 59)** |
| Johannsen et al., 2001  *Adult MLD* | WAIS  Color-Raven AB+B  RAVG Visual Gestalts Test  TMT A + B  Category fluency  Letter fluency  WCST  Clock drawing | RAVG  Not impaired: 16 errors (*z*=-1.5) and 11 errors (*z*=-1.3) | TMT A  Not impaired (*z*= -0.9) | TMT B  Not impaired (*z*= -1.7)  WCST  Not impaired (*z*=  -1.0) | Category fluency  **Impaired: 9 animal words in 1 minute**  Letter fluency  **Impaired: 7 words with ‘s’ in 1 minute** | Color-Raven  Not impaired: 19/24 correct, standardized scores NR  Clock drawing  Not impaired: Numbers and hands placed correctly; Standardized scores NR | Not impaired (FSIQ = 119) |
| Shapiro et al.,1992  *Late infantile MLD* | WPPSI | NI | NI | NI | NI | NI | Not impaired (VIQ = 116; PIQ = 95) |
| Smith et al., 2010  *Adult MLD* | WAIS-R/III  Graded Naming Test | WAIS-R Arithmetic  Not impaired (*z*=  -1.4)  WAIS-R Digit Span  Not impaired (*z* =  -1.8) | NI | NI | WAIS-R Vocabulary  Not impaired (*z*= 0.3)  WAIS-R Similarities  Not impaired (*z*=  -1.0)  Graded Naming Test  Not impaired: 21 out of 30 correct | WAIS-R Block Design  Not impaired (*z*= -1.8) | NR |
| Solders et al., 1998; Solders et al., 2014  *Adult MLD* | WAIS-R  WAIS-III | NI | NI | NI | NI | NI | Patient 1**: Impaired (FSIQ = 52**, VIQ = 62, PIQ = 52)  Patient 2: **Impaired (FSIQ = 62)**  Patient 3: **Impaired (FSIQ = 66)** |
| Videbaek et al., 2021  *Adult MLD* | WAIS-IV | NR | NR | NR | NR | NR | Not impaired (FSIQ = 72, GAI = 72) |
| **Adrenoleukodystrophy (ALD)** | | | | | | | |
| Malm et al., 1997  *CC-ALD* | WISC  NEPSY battery  Trail Making Test  Spiq | Patient 1: NI  Patient 2: ‘Normal’ (NR which test used)  Patient 3: ‘Normal’ (NR which test used) | NI | Trail Making Test  Patient 1: Not impaired (*z*= -1.0)  NEPSY  Patient 2: **Impaired**  **(*z*= -3.0)**  Patient 3: ‘Normal’ (NR which test used) | Spiq  Patient 1: Not impaired (*z*= -1.0)  NEPSY  Patient 2: Not impaired: ‘Normal’ reported  Patient 3: ‘Normal’ (NR which test used) | NEPSY  Patient 2: Not impaired (*z*=  -2.0)  Patient 1: NI  Patient 3: ‘Normal’ (NR which test used) | Patient 1: Not impaired; VIQ = 92 (89-96); PIQ = 85 (82-88); FSIQ = 85 (82-88)  Patient 2: Not impaired; VIQ = 108 (104-111); PIQ = 77 (74-81); FSIQ = 92 (89-96)  Patient 3: Not impaired; VIQ = 130 (≥127); PIQ = 122 (119-126); FSIQ = 119-126) |
| Suzuki et al., 2000  *CC-ALD* | WISC | NI | NI | NI | NI | NI | Patient 1**: Impaired (FSIQ = 60)**  Patient 2: **Impaired (VIQ = 74**; **PIQ = 61**)  Patient 3: NI because severe visual, speech and mental disturbances  Patient 4: NI because severe mental and gait disturbances |
| Turco et al., 2018  *CC-ALD* | WISC-III  Modified Bells Test  WCST  ToL  TNL | NI | NI | Modified Bells Test  Focal attention: Not impaired (*z* =  -0.99)  Sustained attention:  Not impaired (*z* = -1.80)  WCST and ToL  **Impaired**: ‘Impaired planning abilities and notes of impulsiveness’ (Standardized scores NR) | TNL  Receptive language: Not impaired; ‘Normal’ (NR standardized scores) | NI | Not impaired (FSIQ = 77; VIQ = 84; PIQ = 75) |
| **Alexander Disease (AxD)** | | | | | | | |
| Kirsch et al., 2021  *Type I AxD*  *1 patient at four measuring moments* | WISC-IV  WJ-III  Beery  PPVT  EOWPVT  CVLT-C  CMS  CPT-II | CMS  *Visual Immediate*  Age 6: NI  Age 7: Not impaired (*z* = -1.9)  Age 9: NI  Age 12**: Impaired (*z* = -3.3)**  *Visual Delayed*  Age 6: NI  Age 7: Not impaired (*z* = -1.0)  Age 9: NI  Age 12: **Impaired (*z* = -2.5)**  CLVT-C  *Delayed Free Recall*  Age 6: NI  Age 7: Not impaired (*z* = -2.0)  Age 9: **Impaired (*z* = -2.5)**  Age 12: **Impaired (*z* = -4.0)** | NI | CPT-II  (higher T-score = greater impairment)  *Omissions*  Age 6: NI  Age 7: NI  Age 9: **Impaired (T = 84)**  Age 12: **Impaired (T = 77)**  *Commissions*  Age 6: NI  Age 7: NI  Age 9: Not impaired (T = 35)  Age 12: Not impaired (T = 42)  *Hit RT*  Age 6: NI  Age 7: NI  Age 9: **Impaired (T = 91)**  Age 12: **Impaired; (T = 86)** | WJ-III  *LWI*  Age 6: Not impaired (*z* = -1.2)  Age 7: Not impaired (*z* = -0.5)  Age 9: Not impaired (*z* = -1.0)  Age 12: Not impaired (z = -2.0)  *PC*  Age 6: NI  Age 7: Not impaired (*z*= -2.0)  Age 9: **Impaired (*z* = -2.6)**  Age 12: **Impaired (*z*= -3.3)**  *Spelling*  Age 6: **Impaired (*z* = -2.1)**  Age 7: Not impaired (*z* =-1.0)  Age 9: Not impaired (*z* = -1.9)  Age 12: **Impaired(z = -2.5)**  *Writing samples*  Age 6: NI  Age 7: NI  Age 9: **Impaired (*z* = -3.7)**  Age 12: **Impaired (*z* = -5.2)**  PPVT  Age 6: Not impaired (*z* = -0.1)  Age 7: Not impaired (*z* = -1.0)  Age 9: NI  Age 12: **Impaired**  ***(z* = -2.5)**  EOPWT  Age 6: Not impaired (*z* = -0.7)  Age 7: Not impaired (*z* = 0.3)  Age 9: NI  Age 12: Not impaired (*z* = -1.3) | Beery VMI  Age 6: **Impaired (*z* = -2.4)**  Age 7: Not impaired (*z* =  -2.0)  Age 9: NI  Age 12: **Impaired (*z* =**  **-3.7)**  Beery VP  Age 6: Not impaired (*z* =  -1.1)  Age 7: Not impaired (*z* = 0.0)  Age 9: NI  Age 12: **Impaired (*z* =**  **-2.9)** | WISC-IV  Age 6: **Impaired (FSIQ = 68**)  Age 7: **Impaired (FSIQ = 62**)  Age 9: **Impaired (FSIQ = 48**)  Age 12: **Impaired (FSIQ = 43)** |
| Kirsch et al., 2021  *Type II AxD*  *1 patient at three measuring moments* | WISC-IV  WJ-III  Beery  CVLT-C | CVLT-C  *Delayed Free Recall*  Age 12: Not impaired (*z* = 0.0)  Age 13: Not impaired (*z* = 0.5)  Age 16: Not impaired (*z* = -0.5) | NI | NI | WJ-III  *LWI*  Age 12: NI  Age 13: NI Age 16: Not impaired (*z*= -0.3)  *Spelling*  Age 12: Not impaired (*z* = -0.4)  Age 13: NI  Age 16: Not impaired (*z* = 0.2)  *PC*  Age 12: Not impaired (*z* = -0.7)  Age 13: NI  Age 16: Not impaired (*z* = -1.9) | Beery VMI  Age 12: Not impaired (*z* =  -1.0)  Age 13: Not impaired (*z* =  -1.86)  Age 16: **Impaired (*z* =**  **-2.3)**  Beery VP  Age 12: Not impaired (*z* =  -1.33)  Age 13: Not impaired (*z* =  -0.83)  Age 16: Not impaired (*z* =  -1.33) | WISC-IV  Age 12: Not impaired (FSIQ = 78)  Age 13: NI  Age 16: **Impaired (FSIQ = 60)** |
| Restrepo et al., 2011  *Juvenile AxD*  *1 patient at four measuring moments* | WISC-III/WAIS-III  ML-2  HVLT  ROCF  GDS  DKEFS verbal fluency  DKEFS categorical fluency  WCST  BCT  TMT  SDMT  JOLO | ML-2  Age 15: NI  Age 17: Verbal learning; Not impaired (*z* =  -1.6); Delayed verbal; **Impaired (*z* = -2.1)**; Recognition verbal; Not impaired (*z* =  -1.6); Visual learning; Not impaired (*z* =  -0.7); Delayed Visual; Not impaired (*z* =  -1.2)  Age 20: NI  Age 21: NI  HVLT  Age 15: NI  Age 17: NI  Age 20: Learning; **Impaired (*z* =**  **≤-2.60)**; Delayed; **Impaired (*z* =**  **≤-2.60)**; Recognition; **Impaired (*z* =**  **≤-2.60)**  Age 21: Learning; **Impaired (*z* =**  **≤-2.60)**; Delayed; **Impaired (*z* =**  **≤-2.60)**; Recognition; **Impaired (*z* =**  **≤-2.60)**  ROCF  Age 15: NI  Age 17: Copy; **Impaired (*z* =**  **-2.4)**; Learning; Not impaired (*z* =  -1.7); Delayed; **Impaired (*z* =**  **-2.1)**  Age 20: Copy; **Impaired (*z* ≤-2.60)**; Learning; NR; Delayed; Not impaired (*z* =  -1.7)  Age 21: Copy; **Impaired (*z* = ≤-2.60)**; Learning; **Impaired (*z* = ≤-2.60)**; Delayed; **Impaired (*z* = ≤-2.60)** | SDMT Oral  Age 15: NI  Age 17: Not impaired (*z* = 0.1)  Age 20: NI  Age 21: Not impaired (*z* = -1.2) | GDS Vigilance subtest  Age 15: Not impaired (*z* = -0.3)  Age 17: Not impaired (*z* = 0.6)  Age 20: NI  Age 21: Not impaired (*z* = -0.6)  GDS Distractibility Subtest  Age 15: NI  Age 17: NI  Age 20: NI  Age 21: Not impaired (*z* = -1.9)  WCST Perseverative errors  Age 15: **Impaired (*z* = -2.4)**  Age 17: Not impaired (*z* = -0.9)  Age 20: NI  Age 21: Not impaired (*z* = 1.0)  TMT B  Age 15: Not Impaired (*z* = 1.1)  Age 17: Not impaired (*z* = 0.3)  Age 20: NI  Age 21: Not impaired (*z* = -1.4)  DKEFS Sorting  Age 15: NI  Age 17: NI  Age 20: NI  Age 21: **Impaired (*z* = -2.4)**  BCT  Age 15: **Impaired (*z* = -2.1)**  Age 17: **Impaired (*z* = -2.1)**  Age 20: NI  Age 21: NI | Phonemic Fluency  Age 15: NI  Age 17: NI  Age 20: NI  Age 21: Not impaired (*z* = -1.3)  Category Fluency  Age 15: NI  Age 17: NI  Age 20: NI  Age 21: Not impaired (*z* = -1.0) | JOLO  Age 15: NI  Age 17: NI  Age 20: NI  Age 21: Not impaired (*z* =  -1.3)  VMI  Age 15: Not impaired (*z* =  -1.4)  Age 17: NI  Age 20: NI  Age 21: NI | WISC-III  Age 15: Not impaired (FSIQ = 80)  WAIS-III  Age 17: Not impaired (FSIQ = 80)  Age 20: Not impaired (FSIQ = 74)  Age 21: Not impaired (FSIQ = 77) |
| Wilson et al., 2018  *Juvenile AxD* | WISC-R | NI | NI | NI | NI | NI | **Impaired (FSIQ = 60**; VIQ = 65; PIQ = 63) |
|  | | | | | | | |
| **Vanishing White Matter (VWM)** | | | | | | | |
| Trevisan et al., 2021  *Adult VWM* | TMT  SDMT  WCST  Digit Span  RAVLT  BSRT  CDT  Verbal fluency  BNT | Digit Span  Forward; Not impaired, raw score = 6 (Normal value = ≥4.26)  Backward; Not impaired, raw score = 4 (Normal value = ≥2.45)  RAVLT  Immediate recall; Not impaired, raw score = 53 (Normal value = ≥28.56)  Delayed recall; Not impaired, raw score = 9 (Normal value = ≥4.69)  BSRT  Not impaired, raw score = 14.5 (Normal value = ≥8) | TMT A  Not impaired, raw score = 32 (Normal value = ≤59)  Stroop Color  Not impaired, raw score = 40 (Normal value = ≥30.08)  SDMT  Not impaired, raw score = 40 (Normal value = ≥24) | TMT  TMT B; Not impaired, raw score = 126 (Normal value = ≤155)  TMT B A; Not impaired, raw score = 97 (Normal value = ≤90)  Stroop Color Word  Not impaired, raw score = 18 (Normal value = ≥15.8 )  WCST  Total score; Not impaired, raw score = 78 (Normal value = ≤90.5)  Perseverative errors; Not impaired, raw score = 21 (Normal value = ≤42.6)  Non Perseverative errors; Not impaired, raw score = 27 (Normal value = ≤29.9)  Interrupted series; Not impaired, raw score = 2 (Normal value = ≤2-3) | Verbal fluency  Semantic; Not impaired, raw score = 49 (Normal value = ≥25)  Phonological; Not impaired, raw score = 40 (Normal value = ≥17)  BNT  Not impaired, raw score = 52 (Normal value = ≥52) | CDT  Not impaired, raw score = 11 (Normal value = ≥7.5) | NI |
| **Adult-onset Leukoencephalopathy with axonal Spheroids and Pigmented glia (ALSP)** | | | | | | | |
| Mateen et al., 2010  *Adult ALSP* | WAIS-III  TMT  BNT  WMS III  ROCF  JOLO  AVLT | WMS-III  % retention; Not impaired, raw score = 89 (Normal value = 60-71)  AVLT  *Learning 1^st^ trial* Not impaired (z = -1.25)  *Learning 4^th^ trial*  Not impaired (z = -1.35)  *Delayed recall* **Impaired (z = -2.6)** | TMT A  **Impaired (z = -2.40)** | TMT B  Not impaired (z = -1.40) | BNT  Not impaired (z = -0.14) | ROCF Copy  **Impaired (z = ≤-2.60)**  JOLO  **Impaired (z = -2.10)** | Not impaired (VIQ = 110; PIQ = 91) |
| **Krabbe Disease (KD)** | | | | | | | |
| Krivit et al., 1998  *Juvenile KD* | Only domain scores reported, NR which tests are used per domain. | Short term memory  NI  Verbal Learning  Not impaired (z= 0) | Motor speed  **Impaired** (z= -3.3) | NI | Reading  Not impaired (z= 0.1)  Receptive vocabulary  Not impaired (z=-1.1)  Expressive fluency  Not impaired (z= -1.4) | Spatial perception  Not impaired (z = 0.3) | Not impaired (VIQ = 102; Non-verbal IQ = 72) |

*Notes. NI Not Investigated, NR* Not Reported, *FSIQ* Full Scale Intelligence Quotient, *WAIS*  Weschler Adult Intelligence Scale, *TMT* Trail Making Test, *WCST* Wisconsin Card Sorting Test, *WPPSI* Weschler Preschool and Primary Scale of Intelligence, *PIQ* Performance Intelligent Quotient, *VIQ* Verbal Intelligence Quotient, *WISC* Weschler Intelligence Scale for Children, *NEPSY* a Developmental NEuroPSYchological Assessment, *ToL* Tower of London, *TNL* Neuropsychological Lexical Childhood Tests, *WJ-III* Woodstock Johnson Test of Academic Achievement third Edition, *Beery* Beery-Buktenica Developmental Test of Visual Motor Integration, *PPVT* Peabody Picture Vocabulary Test, *EOWPVT* Expressive One Word Picture Vocabulary Test, *CVLT-C* California Verbal Learning Test Childrens Edition, *CMS* Children’s Memory Scale, *CPT-II* Connors Performance Test, second edition, *CMS* Children Memory Scale, *ML-2* Memory and Learning second edition, *HVLT* Hopkins Verbal Learning Test, *ROCF* Rey-Osterrieth Complex Figure Test, *GDS* Gordon Diagnostic System, *SDMT* Symbol Digits Modalities Test, *BCT* Booklet Category Test, *CDT* Clock Drawing Test, *BSRT* Babcock Story Recall Test, *GAI* General Ability Index, *LWI* Letter Word Identification, *PC* Passage Comprehension, , *JOLO* Judgment Of Line Orientation, *BNT* Boston Naming Test, *AVLT* Auditory Verbal Learning Test, *WMS* Weschler Memory Scale.

**Z-scores below the second percentile (i.e. z-scores ≤2.1) were described as impaired.**
