# Supplementary material for: Cognitive and intellectual functioning in leukodystrophy patients: a systematic review
Source: Orphanet J Rare Dis. 2025 Nov 10;20:570. doi: 10.1186/s13023-025-04083-7 (PMC12604172; doi:10.1186/s13023-025-04083-7)
Supplement: Supplementary file 5 — Supplementary Material 5 [file 13023_2025_4083_MOESM5_ESM.docx]

**Additional file 5.** Study characteristics of the included studies on asymptomatic individuals.

**Table 2. Study characteristics of the included studies on asymptomatic individuals**

| **Author (Year)** | **Study Design** | **N [P]/[C]** | **Mean Age**  **Y:M (Range)** | **Gender**  **F:M** | **Criteria for diagnosis** | **Mean age of onset**  **Y:M (range)**  **Distribution subtypes** |
| --- | --- | --- | --- | --- | --- | --- |
| Buermans et al., 2019 | Cross-sectional | 33 [33]/[0] | 44:0 (19-71) | 0:33 | Genetic and MRI analysis | NR  33 Adult |
| Cox et al., 2006 | Cross-sectional | 52 [52]/[0] | 6:7 (2:1-14.6) | 0:52 | Genetic and MRI analysis | NR  52 Childhood Cerebral |
| Furushima et al., 2009 | Longitudinal | 6 [6]/[22] | 10:9 (6:3-14:8) | 0:6 | Genetic and MRI analysis | NR  6 Childhood Cerebral |
| Kaga et al., 2009 | Cross-sectional | 9 [8]/[22] | 9:1 (3:11-14:7) | 0:8 | Genetic and VLFCA analysis | NR  6 Childhood Cerebral |
| Cable et al., 2011; Pierson et al., 2008 | Cross-sectional | 2 [2]/[0] | Patient 1: 6:NR  Patient 2: 4:NR | Patient 1: Male  Patient 2: Female | MRI and urine sulfatide analysis | Patient 1: 6:NR  Patient 2: 4:NR  Juvenile |
| Videbaek et al., 2021 | Longitudinal | 1 [1]/[0] | 17:5 | Male | MRI analysis; Neuropsychological assessment; ARSA deficiency; Genetic analysis | NR  NR |

*Notes. N* Sample size*, P* Patients, *C* Control subjects, *Y* Years*, M* Months*, F* Female*, M* Male, *NR* Not reported, *SD* Standard Deviation, *VLCFA* Very Long Chain Fatty Acids.
