# Supplementary material for: Cognitive and intellectual functioning in leukodystrophy patients: a systematic review
Source: Orphanet J Rare Dis. 2025 Nov 10;20:570. doi: 10.1186/s13023-025-04083-7 (PMC12604172; doi:10.1186/s13023-025-04083-7)
Supplement: Supplementary file 6 — Supplementary Material 6 [file 13023_2025_4083_MOESM6_ESM.docx]

**Additional file 6.** Results of neuropsychological and intellectual assessment reported in the included studies on asymptomatic individuals.

**Table 2.** Results of neuropsychological and intellectual assessment reported in the included studies on asymptomatic individuals

| **Author (Year)** | **Tests** | **Memory** | **Information Processing Speed** | **Attention/ Executive functioning** | **Language** | **Visuospatial functioning & construction** | **Intelligence** |
| --- | --- | --- | --- | --- | --- | --- | --- |
| **Adrenoleukodystrophy (ALD)** | | | | | | | |
| Buermans et al., 2019  *Adult ALD* | Letter fluency  Similarities  Vocabulary  WMS VR  Rey AVLT  Beery VMI  TMT  Stroop  VTS S1 and S3 | Verbal memory  *Rey AVLT IR*  Not impaired (*T*=46.97, *SD* = 8.85)  *Rey AVLT DR*  Not impaired (*T*=49,24, *SD* = 10.05)  *Rey AVLT DR/IR*  Not impaired (*T*=51.52, *SD* = 7.72)  Non-verbal memory  *WMS VR IR*  Not impaired (*T*=50, *SD* = NR)  *WMS VR DR*  Not impaired (*T*=55.44, *SD* = 9.00)  *WMS VR Recognition*  Not impaired (*T*=58, *SD* = NR) | TMT  *A*  Not impaired (*T*=57.29, *SD* = 12.95)  STROOP  *Card I*  Not impaired (*T*=48, *SD* = NR)  *Card II*  Not impaired (*T*=49.94, *SD* = 10.34)  VTS S1  *Reaction Time*  Not impaired (*T*=57.66, *SD* = 12.04)  *Motor speed*  Not impaired (*T*=58.10, *SD* = 11.95)  VTS S3  *Reaction Time*  Not impaired (*T*=46.89, *SD* = 10.72)  *Motor speed*  Not impaired (*T*=51.71, *SD* = 10.92) | TMT  *B*  Not impaired (*T*=53.55, *SD* = 11.18)  *B/A*  Not impaired (*T*=49.81, *SD* = 9.32)  *STROOP*  *Card III*  Not impaired (*T*=53.23, *SD* = 11.14)  *Card III/II*  Not impaired (*T*=55.42, *SD* = 10.59) | Letter fluency  Not impaired (*T*=45.70, *SD* = 8.85)  Similarities  Not impaired (*T*=50 *SD* = NR)  Vocabulary  Not impaired (*T*=47.18 *SD* = 8.00) | Beery VMI  Not impaired (*T*=49, *SD* = NR) | NI |
| Cox et al., 2006  *CC-ALD* | 21 tests in 7 different domains, only domain scores reported. | Not impaired (z = 0.09, SD = 0.70) | NI | Not impaired (z = -0.12, SD = 0.74) | Not impaired (z = 0.06, SD = 0.92) | Not impaired (z = 0.28, SD = 0.83) | Not impaired (FSIQ= 104; VIQ = 101; PIQ = 106) |
| Furushima et al., 2009  *CC-ALD* | WISC III | NI | NI | NI | NI | NI | Not impaired  (mean FSIQ = 94; mean VIQ = 97.5; mean PIQ = 91.5) |
| Kaga et al., 2009  *CC-ALD* | WISC-III or WPPSI  K-ABC GC  Frostig DTVP  RCPM  RCFT | NI | NI | NI | NI | K-ABC GC  Impaired in 3 out of 6 patients; standardized scores NR; 2 missing values  Frostig DTVP  *Constancy of shape*  Impaired in 5 out of 7 patients; standardized scores NR; 1 missing value  *Spacial relationship*  Impaired in 2 out of 7 patients; standardized scores NR; 1 missing value  RCFT  Impaired in 1 out of 6 patients; standardized scores NR; 2 missing values | RCPM  Impaired in 1 out of 6 patients; standardized scores NR; 2 missing values  WISC-III/WPPSI  Not impaired (mean FSIQ = 88; mean VIQ = 92; mean PIQ = 87) |
| **Krabbe Disease (KD)** |  |  |  |  |  |  |  |
| Krivit et al., 1998  *Juvenile KD* | Only domain scores reported, NR which tests are used per domain. | Short term memory  Patient 1: **Impaired** (**z = -2.1**)  Patient 2: Not impaired (z = 1.3)  Verbal Learning  Patient 1: **Impaired** (**z = -2.2**)  Patient 2: NI | Motor speed  Patient 1: **Impaired** (**z = -2.6**)  Patient 2: NI | NI | Reading  Patient 1: **Impaired** (**z = -2.2**)  Patient 2: NI  Receptive vocabulary  Patient 1: Not impaired (z = -0.9)  Patient 2: Not impaired (z = 1.1)  Expressive fluency  Patient 1: Not impaired (z=-0.5)  Patient 2: NI | Spatial Perception  Patient 2: Not impaired (z= -0.9)  Patient 3: NI | Patient 2: Not impaired: VIQ = 95; Non-verbal IQ = 102  Patient 3: Not impaired: VIQ = 116; Non-verbal IQ = 116 |
| **Metachromatic Leukodystrophy** **(MLD)** | | | | | | | |
| Beschle et al., 2020  *Juvenile MLD* | WISC-III/V  WAIS-III/V | NI | NI | NI | NI | NI | Not impaired (FSIQ = 106) |
| Cable et al., 2011; Pierson et al., 2008  *Juvenile MLD* | NR which intelligence test was used | NI | NI | NI | NI | NI | Patient 1: Not impaired; FSIQ = 88  Patient 2: Not impaired; FSIQ = 120 |
| Videbaek et al., 2021  *Adult MLD* | WAIS-IV | NR | NR | NR | NR | NR | Not impaired (FSIQ = 97) |

*Notes. WMS* *VR* Weschler Memory Scale Visual Reproduction, *Rey* *AVLT* Rey Auditory Verbal Learning Test, *Beery VMI* Beery Visual Motor Integration, *TMT* Trail Making Test, *VTS* S1 and S3 Vienna Test System 1 and 3, *IR* Immediate Recall, *SD* Standard Deviation, *DR* Direct Recall, *CC-ALD* Childhood Cerebral Adrenoleukodystrophy, *NI* Not Investigated, *FSIQ* Full Scale Intelligence Quotient, *VIQ* Verbal Intelligence Quotient, *PIQ* Performance Intelligent Quotient, *WISC III* Weschler Intelligence Scale for Children (third edition), *WPPSI* Weschler Preschool and Primary Scale of Intelligence, *K-ABC* (*GC)* Kaufman Assessment Battery for Children (Gestalt Closure), *Frostig DTVP* Frostig Developmental Tets of Visual Perception, *RCPM* Raven Coloured Progressive Matrices, *RCFT* Rey Complex Figure Test, *NR* Not Reported.

**Z-scores below the second percentile (i.e. z-scores ≤2.1) were described as impaired.**
