# Supplementary material for: Cognitive and intellectual functioning in leukodystrophy patients: a systematic review
Source: Orphanet J Rare Dis. 2025 Nov 10;20:570. doi: 10.1186/s13023-025-04083-7 (PMC12604172; doi:10.1186/s13023-025-04083-7)
Supplement: Supplementary file 7 — Supplementary Material 7 [file 13023_2025_4083_MOESM7_ESM.docx]

**Additional file 7.** Risk of bias assessment. The results of the risk of bias assessment for the included group studies according to the Scottish Intercollegiate Guidelines Network (SIGN) quality appraisal checklists and the Joanna Briggs Institute (JBI) critical appraisal checklists.

**Table 1.**

*Risk of bias assessment for cross-sectional (group) studies based on the JBI critical appraisal checklist for analytical cross-sectional studies*

| Author (year) | Description inclusion criteria | Description subjects and setting | Validity of exposure measurement | Validity of measurement of condition | Identification confounding factors | Dealt with confounding factors | Outcome assessment | Statistical analysis | % score | **QUALITY^*^** |
| --- | --- | --- | --- | --- | --- | --- | --- | --- | --- | --- |
| Beschle et al., (2020) | 0 (High) | 1 (Low) | 1 (Low) | 1 (Low) | 0 (High) | 0 (High) | 1 (Low) | 1 (N.A.) | 62,5 | Moderate |
| Bougnères et al., (2021) | 0 (High) | 0 (High) | 1 (Low) | 1 (Low) | 0 (High) | 0 (High) | 1 (Low) | 1 (N.A.) | 50 | Moderate |
| Peters et al., (2004) | 0 (High) | 1 (Low) | 1 (Low) | 1(Low) | 0 (High) | 0 (High) | 1 (Low) | 1 (N.A.) | 62.5 | Moderate |
| Pierpont et al., (2017) | 1 (Low) | 1 (Low) | 1 (Low) | 1 (Low) | 1 (Low) | 1 (Low) | 1 (Low) | 1 (N.A.) | 100 | High |
| Van Rappard et al., (2018) | 0 (High) | 0 (High) | 1 (Low) | 1 (Low) | 1 (Low) | 0 (High) | 1 (Low) | 1 (N.A.) | 62.5 | Moderate |
| Rush et al., (2023) | 0 (High) | 1 (Low) | 1 (Low) | 1 (Low) | 1 (Low) | 0 (High) | 1 (Low) | 1 (N.A.) | 75 | High |
| Schäfer et al., (2021) | 1 (Low) | 1 (Low) | 1 (Low) | 1 (Low) | 1 (Low) | 1 (Low) | 1 (Low) | 1 (N.A.) | 100 | High |
| Strölin et al., (2017) | 0 (High) | 1 (Low) | 1 (Low) | 1 (Low) | 0 (High) | 0 (High) | 1 (Low) | 1 (N.A.) | 62.5 | Moderate |
| Shapiro et al., (2000) | 0 (High) | 1 (Low) | 1 (Low) | 1 (Low) | 1 (Low) | 0 (High) | 1 (Low) | 1 (N.A.) | 75 | High |
| Suzuki et al., (2000) | 0 (High) | 1 (Low) | 0 (High) | 1 (Low) | 1 (Low) | 0 (High) | 1 (Low) | 1 (N.A.) | 62.5 | Moderate |
| Tillema et al., (2015) | 1 (Low) | 1 (Low) | 1 (Low) | 1 (Low) | 0 (High) | 0 (High) | 1 (Low) | 1 (N.A.) | 75 | High |
| Zampini et al., (2023) | 1 (Low) | 1 (Low) | 1 (Low) | 1 (Low) | 0 (High) | 0 (High) | 0 (High) | 1 (N.A.) | 62.5 | Moderate |
| *Notes*. 1 = indicates the article does fulfill the specified criteria or that the criteria is not applicable; 0 = indicates the article does not fulfill the criteria; Low = Low risk of bias; High = High risk of bias, N.A. = Not Applicable, Unclear = Not clear based on provided information in the article.  ^*^Quality scores categorized in three groups: Low: <50%; Moderate 50-70%; High >70% (Dijkshoorn et al., 2021; Kachabian et al., 2024) | | | | | | | | | | |

**Table 2.**

*Risk of bias assessment for case-control (group) studies based on the SIGN checklist for case-control studies*

| Author (year) | Research question | Comparability population of cases and controls | Exclusion criteria | Participation rate | Comparison participants and non-participants | Clear definition of cases | Clear definition of controls | Blinding | Measurement exposure | Confounding | Statistical analysis | % Score | **QUALITY_*_** |
| --- | --- | --- | --- | --- | --- | --- | --- | --- | --- | --- | --- | --- | --- |
| Riva et al., (2000) | 1 (Low) | 1 (Low) | 1 (Low) | 1 (Low) | 1 (Low) | 1 (Low) | 1 (Low) | 1 (D.N.A.) | 1 (Low) | 1 (Low) | 1 (Low) | 100 | High |
| *Notes*. 1 = indicates the article does fulfill the specified criteria or that the criteria is not applicable/does not apply; 0 = indicates the article does not fulfill the criteria; Low = Low risk of bias; High = High risk of bias; D.N.A = Does Not Apply, Can’t say = Not clear based on provided information in the article.  ^*^Quality scores categorized in three groups: Low: <50%; Moderate 50-70%; High >70% (Dijkshoorn et al., 2021; Kachabian et al., 2024) | | | | | | | | | | | | | |

References

1. Beschle J, Döring M, Kehrer C, Raabe C, Bayha U, Strölin M, et al. Early clinical course after hematopoietic stem cell transplantation in children with juvenile metachromatic leukodystrophy. Mol Cell Pediatr. 2020;7(1).

2. Bougnères P, Hacein-Bey-Abina S, Labik I, Adamsbaum C, Castaignède C, Bellesme C, Schmidt M. Long-Term Follow-Up of Hematopoietic Stem-Cell Gene Therapy for Cerebral Adrenoleukodystrophy. Hum Gene Ther. 2021;32(19):1260-9.

3. Peters C, Charnas LR, Tan Y, Ziegler RS, Shapiro EG, DeFor T, et al. Cerebral X-linked adrenoleukodystrophy: The international hematopoietic cell transplantation experience from 1982 to 1999. Blood. 2004;104(3):881-8.

4. Pierpont EI, Eisengart JB, Shanley R, Nascene D, Raymond GV, Shapiro EG, et al. Neurocognitive Trajectory of Boys Who Received a Hematopoietic Stem Cell Transplant at an Early Stage of Childhood Cerebral Adrenoleukodystrophy. JAMA Neurol. 2017;74(6):710-7.

5. van Rappard DF, de Vries ALC, Oostrom KJ, Boelens JJ, Hollak CEM, van der Knaap MS, Wolf NI. Slowly Progressive Psychiatric Symptoms: Think Metachromatic Leukodystrophy. J Am Acad Child Adolesc Psychiatry. 2018;57(2):74-6.

6. Rush BK, Tipton PW, Strongosky A, Wszolek ZK. Neuropsychological profile of CSF1R-related leukoencephalopathy. Front Neurol. 2023;14:1155387.

7. Schäfer L, Roicke H, Fischer M, Sühnel A, Köhler W. Cognitive functions in adult‐onset phenotypes of X‐linked adrenoleukodystrophy. Schäfer, Lisa: Department of Neurology, Leipzig University Medical Center, Leukodystrophy Outpatient Clinic, Liebigstrasse 20a, Leipzig, Germany, 04103, lisa.schaefer@medizin.uni-leipzig.de US: John Wiley & Sons; 2021 2021. 266-73 p.

8. Strolin M, Krageloh-Mann I, Kehrer C, Wilke M, Groeschel S. Demyelination load as predictor for disease progression in juvenile metachromatic leukodystrophy. Ann Clin Transl Neurol. 2017;4(6):403-10.

9. Shapiro E, Krivit W, Lockman L, Jambaque I, Peters C, Cowan M, et al. Long-term effect of bone-marrow transplantation for childhood-onset cerebral X-linked adrenoleukodystrophy. Lancet. 2000;356(9231):713-8.

10. Suzuki Y, Isogai K, Teramoto T, Tashita H, Shimozawa N, Nishimura M, et al. Bone marrow transplantation for the treatment of X-linked adrenoleukodystrophy. J Inherit Metab Dis. 2000;23(5):453-8.

11. Tillema JM, Derks MG, Pouwels PJ, de Graaf P, van Rappard DF, Barkhof F, et al. Volumetric MRI data correlate to disease severity in metachromatic leukodystrophy. Ann Clin Transl Neurol. 2015;2(9):932-40.

12. Zampini L, Draghi L, Zanchi P. Developmental Profiles in Children and Young Adults with Alexander Disease. Dev Neurorehabil. 2023;26(4):253-61.

13. Riva D, Bova SM, Bruzzone MG. Neuropsychological testing may predict early progression of asymptomatic adrenoleukodystrophy. Neurology. 2000;54(8):1651-5.
